# Acute respiratory failure in immunocompromised patients: outcome and clinical features according to neutropenia status

**DOI:** 10.1186/s13613-020-00764-7

**Published:** 2020-10-22

**Authors:** Djamel Mokart, Michael Darmon, Peter Schellongowski, Peter Pickkers, Marcio Soares, Jordi Rello, Philippe R. Bauer, Andry van de Louw, Virginie Lemiale, Fabio Silvio Taccone, Ignacio Martin-Loeches, Jorge Salluh, Katerina Rusinova, Sangeeta Mehta, Massimo Antonelli, Achille Kouatchet, Andreas Barratt-Due, Miia Valkonen, Precious Pearl Landburg, Ramin Brandt Bukan, Frédéric Pène, Victoria Metaxa, Gaston Burghi, Colombe Saillard, Lene B. Nielsen, Emmanuel Canet, Magali Bisbal, Elie Azoulay, for the Efraim investigators and the Nine-I study group

**Affiliations:** 1 Réanimation Polyvalente Et Département D’Anesthésie Et de Réanimation, Institut Paoli-Calmettes, 232 Bd Sainte Marguerite 13009, Marseille Cedex 09, France; 2 Medical Intensive Care Unit, APHP, Hôpital Saint‑Louis, Famirea Study Group, ECSTRA Team and Clinical Epidemiology, UMR 1153, Center of Epidemiology and Biostatistics, Sorbonne Paris Cité, CRESS, INSERM, Paris Diderot Sorbonne University, Paris, France; 3 Department of Medicine I, Medical University of Vienna, Vienna, Austria; 4 The Department of Intensive Care Medicine (710), Radboud University Medical Center, Nijmegen, The Netherlands; 5 The Department of Critical Care and Graduate Program in Translational Medicine, D’Or Institute for Research and Education, Programa de Pós-Graduação Em Clínica Médica, Rio de Janeiro, Brazil; 6 CIBERES, Instituto de Salud Carlos III, Barcelona, Spain; 7 Clinical Research/Epidemiology In Pneumonia and Sepsis (CRIPS), Vall d’Hebron Institute of Research (VHIR), Barcelona, Spain; 8 Anesthesiology Department, Clinical Research in ICU, CHU Nîmes, University Montpellier, Nîmes, France; 9 Pulmonary and Critical Care Medicine, Mayo Clinic, Rochester, MN USA; 10 Division of Pulmonary and Critical Care, Penn State University College of Medicine, Hershey, PA USA; 11 Department of Intensive Care, Hôpital Erasme, Université Libre de Bruxelles (ULB), Brussels, Belgium; 12 Department of Intensive Care Medicine, Multidisciplinary Intensive Care Research Organization (MICRO), St. James’s Hospital, Dublin, Ireland; 13 Department of Clinical Medicine, Trinity College, Wellcome Trust‑HRB Clinical Research Facility, St James Hospital, Dublin, Ireland; 14 Department of Anesthesiology and Intensive Care Medicine and Institute for Medical Humanities, 1St Faculty of Medicine, Charles University in Prague and General University Hospital, Prague, Czech Republic; 15 Department of Medicine and Interdepartmental Division of Critical Care Medicine, Sinai Health System, University of Toronto, Toronto, ON Canada; 16 Dept of Anesthesia Intensive Care and Emergency Medicine, Fondazione Policlicnico Universitario A.Gemelli IRCCS. Università Cattolica del Sacro Cuore, Rome, Italy; 17 Department of Medical Intensive Care Medicine, University Hospital of Angers, Angers, France; 18 Department of Emergencies and Critical Care, Oslo University Hospital, Oslo, Norway; 19 Division of Intensive Care Medicine, Department of Anesthesiology, Intensive Care and Pain Medicine, University of Helsinki and Helsinki University Hospital, Helsinki, Finland; 20 Department of Critical Care, University Medical Center Groningen, Groningen, The Netherlands; 21 Department of Anesthesiology I, Herlev University Hospital, Herlev, Denmark; 22 Medical ICU, Cochin Hospital, Assistance Publique-Hôpitaux de Paris and University Paris Descartes, Paris, France; 23 King’s College Hospital, London, SE5 9RS UK; 24 Terapia Intensiva, Hospital Maciel, Montevideo, Uruguay; 25 Intensive Care Department, University of Southern Denmark, Odense, Denmark; 26 Department of Anaesthesia and Intensive Care, Odense University Hospital, Odense, Denmark; 27 Medical Intensive Care Unit, Hôtel Dieu-HME University Hospital of Nantes, Nantes, France

## Abstract

**Background:**

The impact of neutropenia in critically ill immunocompromised patients admitted in a context of acute respiratory failure (ARF) remains uncertain. The primary objective was to assess the prognostic impact of neutropenia on outcomes of these patients. Secondary objective was to assess etiology of ARF according to neutropenia.

**Methods:**

We performed a post hoc analysis of a prospective multicenter multinational study from 23 ICUs belonging to the Nine-I network. Between November 2015 and July 2016, all adult immunocompromised patients with ARF admitted to the ICU were included in the study. Adjusted analyses included: (1) a hierarchical model with center as random effect; (2) propensity score (PS) matched cohort; and (3) adjusted analysis in the matched cohort.

**Results:**

Overall, 1481 patients were included in this study of which 165 had neutropenia at ICU admission (11%). ARF etiologies distribution was significantly different between neutropenic and non-neutropenic patients, main etiologies being bacterial pneumonia (48% vs 27% in neutropenic and non-neutropenic patients, respectively). Initial oxygenation strategy was standard supplemental oxygen in 755 patients (51%), high-flow nasal oxygen in 165 (11%), non-invasive ventilation in 202 (14%) and invasive mechanical ventilation in 359 (24%). Before adjustment, hospital mortality was significantly higher in neutropenic patients (54% vs 42%; *p* = 0.006). After adjustment for confounder and center effect, neutropenia was no longer associated with outcome (OR 1.40, 95% CI 0.93–2.11). Similar results were observed after matching (52% vs 46%, respectively; *p* = 0.35) and after adjustment in the matched cohort (OR 1.04; 95% CI 0.63–1.72).

**Conclusion:**

Neutropenia at ICU admission is not associated with hospital mortality in this cohort of critically ill immunocompromised patients admitted for ARF. In neutropenic patients, main ARF etiologies are bacterial and fungal infections.

## Introduction

Therapeutic advances in oncology and hematology have led to improved survival in patients with cancer [1–3], particularly in the sickest subgroups of patients treated with mechanical ventilation or vasopressors [4]. This effect seems to be also pronounced for neutropenic patients. Neutropenia is present in approximately one-third of critically ill cancer patients. Neutropenia is a complex time-dependent [5] and a biphasic immunosuppression state in which the period of neutropenia and neutropenia recovery represents high-risk time for sepsis, acute respiratory failure (ARF), use of stimulating factors (e.g., G-CSF), pre-engraftment and engraftment syndromes [6]. Prognostic impact of neutropenia remains controversial, particularly in high-risk situations such as ARF, as there are sparse data on critically ill immunocompromised population [7–11]. In immunocompromised patients with ARF, failure to identify etiological diagnosis is associated with worse outcome [8, 12]. A standardized approach (the DIRECT approach) can be used to assess the cause of ARF [13–15]. Using this tool, previous studies suggest neutropenic patients with ARF have a high risk of bacterial or fungal infection when compared to other immune defects [15]. These data are, however, recovered in a single-center study and external validity of these findings is needed.

The primary objective of this study was to assess prognostic impact of neutropenia on hospital mortality in critically ill patients with immune defect and ARF. Secondary objective was to assess whether neutropenia was associated with specific etiologies.

## Patients and methods

This study is a preplanned ancillary analysis of the prospective multicenter multinational Efraim study [8] coordinated by the Grrr-OH (Groupe de Recherche en Réanimation Respiratoire en Onco-Hématologie) and conducted by the Nine-I (Caring for critically ill immunocompromised patients) network. Initially, 1611 immunocompromised adults admitted to the ICU for ARF were included from 68 ICUs in 16 countries [8]. After IRB approval, each participating ICU prospectively included patients between November 2015 and July 2016. Inclusion criteria were age (≥ 18 years), acute hypoxemic respiratory failure (PaO_2_ < 60 mmHg or SpO_2_ < 90% on room air, or tachypnea > 30/min, or labored breathing or respiratory distress or dyspnea at rest or cyanosis), need for more than 6 L/min oxygen, respiratory symptom duration less than 72 h and non-AIDS-related immune deficiency defined as hematologic malignancy or solid tumor (active or in remission for less than 5 years, including recipients of autologous or allogeneic stem cell transplantation), solid organ transplant, long-term (> 30 days) or high-dose (> 1 mg/kg/day) steroids, or any immunosuppressive drug for more than 30 days. Patients with postoperative acute respiratory failure (within 6 days of surgery), those admitted after a cardiac arrest, patients admitted only to secure bronchoscopy, and patients/surrogates who declined study participation were not included. Patients were included in this analysis if leucocytes count on ICU admission was available and if hospital mortality was reported. Neutropenia was defined as a neutrophil count (or if missing as a white blood cell count) lower than 1 G/L at ICU admission. A patient was considered to be neutropenic only if he had neutropenia at ICU admission.

### Data collection

Patient demographic, immunologic (oncologic, hematologic, drugs, etc.), neutropenia, hematopoietic stem cell transplant, others comorbidities, functional status (ECOG performance status—Eastern Cooperative Oncology Group), ARF details (cause, diagnostic investigations, initial oxygen strategy—non-invasive ventilation [NIV], high-flow nasal oxygen [HFNO], standard oxygen therapy, invasive mechanical ventilation, and ARF management), critical care treatments and outcomes were collected by each participating institution, as previously described [7, 8]. All management decisions were made by the clinical teams of each institution according to their standard of practice. All diagnoses were reviewed by two study investigators for coherence and for alignment with established definitions [8].

### Statistical analysis

Quantitative variables were described as median (interquartile range [IQR]) and were compared between groups using the non-parametric Wilcoxon rank-sum test. Qualitative variables were described as frequency (percentages) and were compared between groups using Fisher’s exact test.

Hierarchical models were used to assess factors independently associated with hospital mortality. First, logistic regression was performed for variable selection. We used conditional stepwise regression with 0.2 as the critical *p* value for entry into the model, and 0.1 as the *p* value for removal. It was planned a priori to test influence of neutropenia in the final model, and even if this variable had not been selected. Interactions and correlations between the explanatory variables were carefully checked. Continuous variables for which log-linearity was not confirmed were transformed into categorical variables according to median or IQR. The final models were assessed by calibration, discrimination, and relevancy. Residuals were plotted, and the distributions inspected. A hierarchical model was then performed using variables previously selected along with center as random effect on the intercept. This model adjusting for clustering effect was planned a priori to be main result of the analysis. Same validation methods were used as previously. Adjusted odds ratios (OR) of variables present in the final model are presented with their 95% confidence intervals.

In a sensitivity analysis, a double adjustment was performed. First a matching was performed according to risk factor to exhibit neutropenia at ICU admission. A propensity score (PS) matched analysis was conducted comparing neutropenic to non-neutropenic patients. Variables for t the PS model were selected according to their statistical association with neutropenia (*p* value entry threshold < 0.2) and included age, gender, immune defect, hematopoietic stem cell transplantation (HSCT), kidney comorbidity, ARF diagnosis, mechanical ventilation, use of vasopressors, and renal replacement treatment. Case-matching was conducted using a 1:1 matching procedure without replacement and according to the nearest neighbor method. Adequacy of the matching procedure was assessed by plotting PS across two groups and then assessing differences across groups for considered variables using standardized mean difference. Univariate analysis was performed and a logistic regression model was then performed, including variables that matched poorly or were unmatched [standardized mean difference (SMD) above 0.5]. A mixed model was then performed using unmatched variables associated with mortality in the matched cohort, center being included as a random effect on the intercept. As a sensitivity analysis, this analysis was run forcing variables with SMD above 0.2 in the model.

Kaplan–Meier graphs were used to express the probability of death from inclusion to hospital discharge, censored at day 90. Influence of neutropenia status was assessed by the log-rank test. Statistical analyses were performed with R statistical software, version 3.4.3 (available online at https://www.r-project.org/) and packages ‘Survival’, ‘MatchIt’, ‘lme4’, and ‘lmerTest’. A *p* value < 0.05 was considered significant.

## Results

**Fig. 1:**
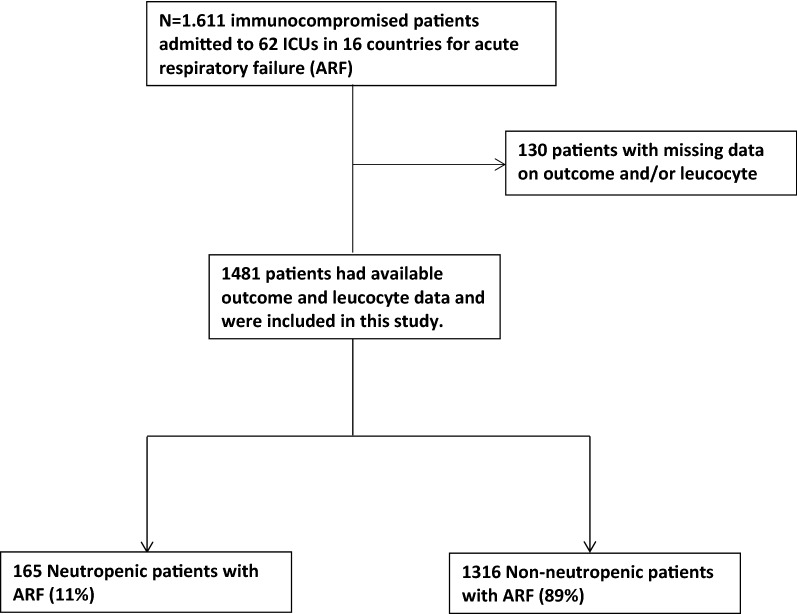
Study flowchart

Overall, among the 1611 patients included in the EFRAIM study, 1481 patients had mortality and white cell count data available and were included in this study (Fig. 1). Median age was 64 years [IQR 55–72] and 613 patients (39.7%) were female. Median SOFA score was 7 [4–10] and performance status was 1 [0–3]. The most common immune defect was hematological malignancy (HM) in 533 patients (36%), solid tumor in 473 (32%), systemic disease in 155 (10.5%), and the use of immunosuppressive drugs in 117 (8%). Neutropenia at admission was observed in 165 patients (11%). Among neutropenic patients, more than 95% (*n* = 157) of the patients presented at ICU admission with profound neutropenia (neutrophils < 0.5 G/L) and 93% (*n* = 153) with severe neutropenia (neutrophils < 0.1 G/L). Initial oxygenation strategy was standard oxygen in 755 patients (51%), high-flow nasal oxygen in 165 (11%), non-invasive mechanical ventilation in 202 (14%), and invasive mechanical ventilation in 359 (24%). At ICU admission neutropenic patients presented with a higher SOFA score than non-neutropenic patients (10 (7–12) vs 6 (4–10), respectively, *p* < 0.001). Neutropenic patients were more frequently treated with vasopressors than non-neutropenic patients (117 (70.9%) vs 732 (55.6%), respectively, *p* < 0.001), with renal replacement therapy (RRT) (43 (26.1%) vs 208 (15.8%), respectively, *p* = 0.001), and distribution of respiratory support at ICU admission was also different between groups (*p* = 0.017, Table 1). During ICU stay, cumulative incidence of invasive mechanical ventilation was not different between groups (*p* = 0.28, Additional file 1: Fig. S1). Before adjustment, hospital mortality rate was significantly higher in neutropenic patients (54% vs 42% in non-neutropenic patients; *p* = 0.006) (Table 1). After adjustment for confounders and center effect, neutropenia was no longer associated with outcome (OR 1.40, 95% CI 0.93–2.11), RRT, vasopressor use, and older age being independently associated with higher hospital mortality (Additional file 2: Table S1; Hosmer–Lemeshow goodness of fit: *p* = 0.12; AUC 0.72; 95% CI 0.70–0.74).

**Table 1:** Characteristics of neutropenic vs non-neutropenic patients

	Non-neutropenic (*n* = 1316)	Neutropenic (*n* = 165)	*p* value
Age (median [IQR])	65 (55–72)	59 (45.5–67)	< 0.001
Female	529 (40.5)	56 (34.1)	0.14
SOFA (median [IQR])	6 (4–10)	10 (7–12)	< 0.001
ECOG-PS (median [IQR])	1 (1–2)	1 (1–2)	0.51
Organ transplantation	125 (10.6)	3 (1.9)	0.001
Leucocytes count at ICU admission(G/L)	13 (6–27)	0.00 (0.00–0.00)	< 0.001
Neutrophils count at ICU admission(G/L)	10(4–23)	0.00 (0.00–0.00)	< 0.001
Hematopoietic cell transplant (HCT)			< 0.001
No HCT	1139 (86.6)	107 (64.8)	
Autologous HCT	68 (5.2)	26 (15.8)	
Allogeneic HCT	109 (8.3)	32 (19.4)	
Immune defect			< 0.001
Acute leukemia	167 (12.7)	70 (42.4)	
Chronic leukemia	68 (5.2)	6 (3.6)	
Hodgkin disease	28 (2.1)	5 (3)	
Drug-induced immunosuppression	105 (8)	2 (1.2)	
Myeloma	122 (9.3)	13 (7.9)	
Non-Hodgkin lymphoma	138 (10.5)	33 (20)	
Other	103 (7.8)	16 (9.7)	
Solid tumor	433 (32.9)	19 (11.5)	
Systemic	152 (11.6)	1 (0.6)	
Comorbidities			
Diabetes	266 (20.8)	17 (10.8)	0.004
Kidney	209 (16.2)	9 (5.6)	0.001
Cirrhosis	48 (3.7)	4 (2.5)	0.57
Respiratory support			0.02
High-flow nasal oxygen	138 (10.5)	27 (16.4)	
Non-invasive ventilation	182 (13.8)	20 (12.1)	
O_2_	664 (50.5)	91 (55.2)	
Mechanical ventilation	332 (25.2)	27 (16.4)	
Vasopressors	732 (55.6)	117 (70.9)	< 0.001
Renal replacement therapy	208 (15.8)	43 (26.1)	0.001
Acute respiratory failure diagnosis (%)			< 0.001
Bacterial	356 (27.1)	79 (47.9)	
Fungal	74 (5.6)	17 (10.3)	
Other	691 (52.5)	53 (32.1)	
Pneumocystis	48 (3.6)	3 (1.8)	
Unknown	147 (11.2)	13 (7.9)	
No BAL use	823 (62.5)	108 (65.5)	0.52
ICU mortality	417 (31.7)	72 (43.6)	0.003
Hospital mortality	557 (42.2)	89 (54)	0.006

*SOFA* Sequential Organ Failure Assessment score, *ECOG-PS* Eastern Cooperative Oncology Group-Performance Status, *BAL* bronchoalveolar lavage, *ICU* intensive care unit

**Fig. 2:**
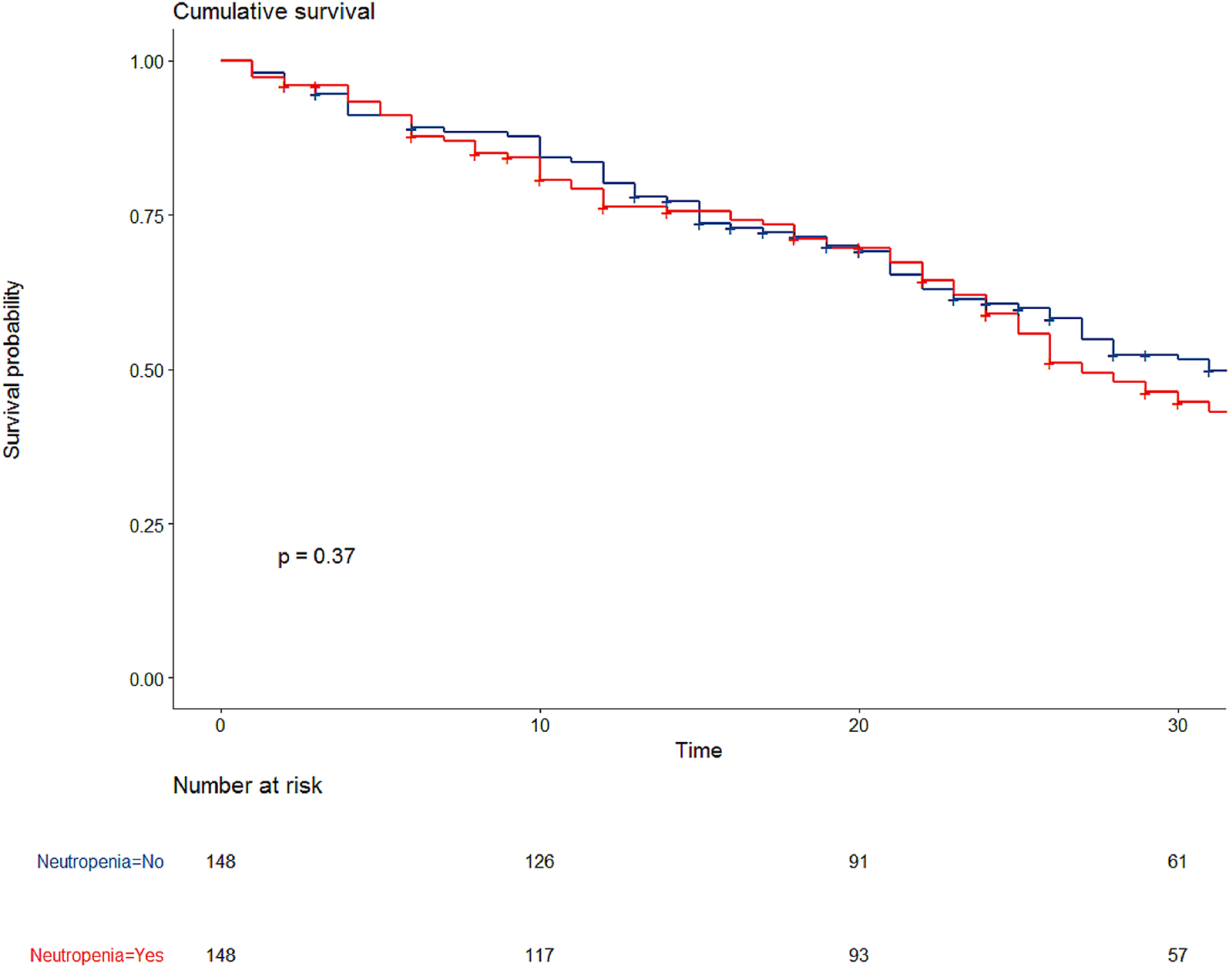
Hospital survival after propensity score matching comparing 148 neutropenic patients with 148 non-neutropenic patients (Kaplan–Meier curve)

After propensity score (PS) matching, 148 patients in each cohort were compared (Table 2, Fig. 2). Standardized mean differences suggest adequate adjustment on considered variables except for stem cell transplantation (Additional file 3: Fig. S2, SMD 0.25). Hospital mortality in the matched cohort did not differ between neutropenic and non-neutropenic patients (52% vs 46%, respectively; *p* = 0.35) (Table 2, Fig. 2).

**Table 2:** Characteristics of neutropenic vs non-neutropenic patients after propensity score matching

	Non-neutropenic (*n* = 148)	Neutropenic (*n* = 148)	*p* value
Age (median [IQR])	55.55 (15.85)	55.75 (14.12)	0.91
Female (%)	54 (36.5)	49 ( 33.1)	0.63
SOFA (median [IQR])	8.00 [5.00, 10.00]	10.00 [7.00, 12.00]	< 0.001
Hematopoietic cell transplant (HCT)			0.07
No (HCT)	107 (72.3)	97 (65.5)	
Autologous HCT	12 (8.1)	25 (16.9)	
Allogeneic HCT	29 (19.6)	26 ( 17.6)	
Immune defect (ID)			0.94
Acute leukemia	66 (44.6)	62 (41.9)	
Chronic leukemia	2 (1.4)	6 (4.1)	
Hodgkin disease	7 (4.7)	5 (3.4)	
Drug-induced immunosuppression	1 (0.7)	1 (0.7)	
Myeloma	13 (8.8)	13 (8.8)	
Non-Hodgkin lymphoma	31 (20.9)	32 (21.6)	
Other	10 ( 6.8)	11 (7.4)	
Solid tumor	16 (10.8)	17 (11.5)	
Systemic	2 ( 1.4)	1 (0.7)	
Kidney comorbidity	13 ( 8.8)	9 (6.1)	0.51
Respiratory support			0.73
High-flow nasal oxygen	28 (18.9)	24 (16.2)	
Non-invasive ventilation	13 ( 8.8)	16 (10.8)	
O2	78 (52.7)	84 (56.8)	
Mechanical ventilation	29 (19.6)	24 (16.2)	
Vasopressors	115 (77.7)	103 (69.6)	0.15
Renal replacement therapy	42 (28.4)	39 (26.4)	0.79
Acute respiratory failure diagnosis			0.82
Bacterial	69 (46.6)	73 (49.3)	
Fungal	15 (10.1)	16 (10.8)	
Other	49 (33.1)	44 (29.7)	
Pneumocystis	1 ( 0.7)	3 (2.0)	
Unknown	14 ( 9.5)	12 (8.1)	
No BAL use	77 (52.0)	95 (64.2)	0.05
Hospital mortality	68 (45.9)	77 (52.0)	0.35

*SOFA* Sequential Organ Failure Assessment score, *BAL* bronchoalveolar lavage

In the analysis adjusted for confounders and center effect, there was no association between neutropenia and hospital survival (OR 1.04; 95% CI 0.63–1.72; Additional file 4: Table S2; Hosmer–Lemeshow goodness of fit: *p* = 0.58; AUC 0.68; 95% CI 0.61–0.74).

Acute respiratory failure etiologies distribution was significantly different between neutropenic and non-neutropenic patients, main etiologies being bacterial pneumonia (48% vs 27%), invasive fungal infection (10% vs 7%), and *Pneumocystis jiroveci* pneumonia (2% vs 4%), other diagnosis (32% vs 56%), and undetermined etiology (8% vs 11%), respectively (p < 0.001) (Table 1, Additional file 5: Fig. S3). Microbiologically documented infection also displayed a different profile in neutropenic patients (Table 3, *p* = 0.04). In addition, influenza (15%) or non-influenza (28%) viral infections as well as cardiogenic edema (24%) appeared to be frequent other situations in neutropenic patients, whereas tumor infiltrations were rarely diagnosed (4%) (Additional file 6: Table S3).

**Table 3:** Bacterial infectious diagnoses

	Non-neutropenic (*n* = 356)	Neutropenic (*n* = 79)	*p *value
			0.044
Gram-negative bacteria			
*Pseudomonas*	31 (9%)	14 (18%)	
*Klebsiella*	33 (9%)	14 (18%)	
*Escherichia coli*	40 (11%)	14 (18%)	
*Enterobacter*	18 (5%)	3 (4%)	
*Stenotrophomonas*	3 (1%)	3 (4%)	
*Legionella*	4 (1%)	2 (3%)	
*Branhamella catarrhalis*	6 (2%)	2 (3%)	
*Acinetobacter*	7 (2%)	1 (1%)	
*Haemophilus influenzae*	13 (4%)	1 (1%)	
*Campylobacter jejuni*	0 (0%)	1 (1%)	
*Citrobacter*	1 (0.5%)	1 (1%)	
*Proteus*	4 (1%)	0	
*Hafnia alvei*	3 (1%)	0	
Morganella	3 (1%)	0	
*Serratia*	3 (1%)	0	
*Salmonella*	2 (0.5%)	0	
*Neisseria meningitidis*	2 (0.5%)	0	
*Bacteroides*	1 (0.5%)	0	
*Bordetella hinzii*	1 (0.5%)	0	
Gram-positive bacteria			
*Coagulase-negative staphylococci*	49 (14%)	9 (11%)	
*Enterococcus*	31 (9%)	6 (8%)	
*Staphylococcus aureus*	49 (14%)	4 (5%)	
*Streptococcus*	11 (3%)	2 (3%)	
*Streptococcus pneumoniae*	34 (10%)	1 (1%)	
*Actinomyces*	3 (1%)	0	
*Clostridium*	1 (0.5%)	0	
Others			
Mycoplasma	3 (1%)	0	

## Discussion

In this large cohort study, neutropenia was not associated with outcome in critically ill immunocompromised patients with ARF after adjustment for confounders. Etiology of ARF was however significantly different between neutropenic and non-neutropenic patients, with neutropenia being associated with a higher rate of bacterial and fungal infection.

Although neutropenia remains associated with a poor outcome in general ICU populations [16], several recent studies have not shown an association between neutropenia and outcomes of critically ill cancer patients [7, 17]. Neutropenia remains an accepted side effect of most treatments administered to hematological patients [18]. Neutropenia is also associated with various complications including severe sepsis [19, 20], acute respiratory failure [21], and specific adverse effects such as neutropenic enterocolitis [22]. Although these side effects are likely to influence the outcome of critically ill patients, results of studies in this field remain controversial. In a recent multicenter observational study including 289 critically ill neutropenic cancer patients, the hospital mortality rate was 55%, however neutropenia was not associated with outcome after adjustment for confounder [10]. Independent factors associated with hospital mortality were age, allogeneic HSCT, invasive mechanical ventilation, RRT, microbiologic documentation; whereas, neutropenic enterocolitis was associated with survival. In contrast, a recent individual patient data meta-analysis including 7512 critically ill cancer patients concluded that neutropenia was independently associated with increased risk of death of 10% [4]. In the present study, neutropenia was not associated with outcome after adjustment for confounders and propensity score analysis. Importantly, after matching, neutropenia, when associated with acute respiratory failure, was not a risk factor for hospital mortality regardless of the underlying immunosuppression. However, crude hospital mortality was 54% underlining that neutropenia and acute respiratory failure were associated with a high morbidity and mortality.

Neutropenia was found to be associated with higher severity and rate of organ dysfunction. This might be related to a longer delay to ICU admission [23]. Hence, although HFNO was used more frequently in neutropenic patients at ICU admission, use of invasive mechanical ventilation was found to be required similarly in neutropenic and non-neutropenic patients during ICU stay. Whether the presence of neutropenia at ICU admission might modify treatment strategy during ICU may therefore deserve to be further evaluated.

An important point of the Efraim study was the association between of undiagnosed etiology of ARF and higher hospital mortality [8]. In our study, we found a low rate of undetermined etiological diagnosis in the subgroup of neutropenic patients (8%), identification of etiology being achieved mainly by non-invasive diagnostic strategies. This highlights the importance of a relevant diagnostic work-up for high-risk patients admitted to ICU occurring in specialized centers. During neutropenia, phagocytosis, chemotaxis and the oxidative capacities of granulocytes are affected thus predisposing patients to bacterial infections and invasive fungal infections (IFI) [14]. Our study showed, in a selected population of immunosuppressed patients admitted to ICU for ARF with neutropenia, the main diagnosis was bacterial and/or fungal infections. In neutropenic patients, *non-fermenting Gram-negative bacilli* such as *Pseudomonas* as well as *enterobacteriaceae* such as *Klebsiella* were frequently documented in contrast to *Staphylococcus aureus* infections [24]. Our results are strongly in accordance with the DIRECT approach, in which the "I" designates the type of immunosuppression and represents an essential step in patient management as it suggests for neutropenic patients, first to preferentially suspect bacterial or fungal infections [13, 14, 24], second to encourage a non-invasive diagnostic strategy with bronchoalveolar lavage used only in selected group of patients [25], and third to start empirical anti-microbial treatment targeting non-fermenting Gram-negative bacilli [13, 20, 24] and/or the most frequent IFIs such as invasive pulmonary aspergillosis [26]. The detection of respiratory viruses in the upper airway is common in critically ill hematologic patients [27]. In patients with ARF, respiratory virus detection was independently associated with ICU mortality [27]. Interestingly, influenza viral infection in immunosuppressed patients is also associated with ICU mortality [28]. In neutropenic patients, the viral risk is currently unknown. We have shown in this study that viral infections were also frequent in neutropenic patients with ARF, however molecular multiplex respiratory virus techniques are not standardized [27]. The clinical implications of these results remain not only to be evaluated in terms of empiric anti-microbial treatment, but also in terms of diagnostic strategy [25, 29].

This study has several limitations. First, neutropenia being a time-dependent parameter, this characteristic was evaluated at ICU admission to avoid influence of competing events on findings. Along this line, the impact of neutropenia recovery on changes in respiratory status could not be assessed due to the lack of data on this point. Although our results are robust with regard to the prognostic influence of neutropenia at ICU admission, no conclusion can be drawn with regard to the prognostic influence of neutropenia occurring during ICU stay. In this line, influence of neutropenia duration was not assessed either before ICU admission or during ICU stay and could have influenced our findings. In addition, management of neutropenic patients may vary across centers and some center-specific management strategies or ICU admission policies might have influenced our findings. However, the analysis was performed using a hierarchical model that should have partly adjusted for clustering effect. Unfortunately, the ratio of patients who died in a context of therapeutic limitation was not available in our data collection. However, the majority of neutropenic patients admitted to intensive care are admitted in a context of recent chemotherapy, in which context an ICU full code management strategy is often implemented. Lastly, lack of statistical power may have explained the lack of association between neutropenia and outcome [30, 31]. Our findings were, however, robust and persistent after sensitivity analysis suggesting that the impact of neutropenia on the prognosis was less than the degree of organ dysfunction and severity or underlying etiology of ARF.

## Conclusion

Neutropenia at ICU admission is not associated with hospital mortality in this cohort of critically ill immunocompromised patients admitted for ARF. In neutropenic patients, the main causes of ARF were bacterial and fungal infections.

## Supplementary information

**Additional file 1: Fig. S1.** Cumulative incidence of invasive mechanical ventilation during ICU stay according to neutropenia status while taking into account competing risk of mortality and discharge from ICU (Gray test: *p* = 0.28).

**Additional file 2: Table S1.** Final mixed selected model with neutropenia and centre effect.

**Additional file 3: Fig. S2.** Change in standardized mean difference after matching.

**Additional file 4: Table S2.** Hierarchical model assessing factors associated with hospital mortality in the matched cohort. Center effect was entered as random effect on the intercept.

**Additional file 5: Fig. S3.** Main etiologies of ARF in the overall population, 1316 non-neutropenic patients vs 165 neutropenic patients.

**Additional file 6: Table S3.** Other diagnoses (main etiologies).

## Data Availability

The datasets generated and analyzed during the current study are not publicly available due to the potential risk of leakage of personally identifiable information.

## References

[1] Brenner H (2002). Long-term survival rates of cancer patients achieved by the end of the 20th century: a period analysis. Lancet.

[2] van Vilet M, van der Burgt MP, van der Velden WJ, van der Hoeven JG, de Haan AF, Donnelly JP (2014). Trends in the outcomes of Dutch haematological patients receiving intensive care support. Neth J Med.

[3] Verdecchia A, Francisci S, Brenner H, Gatta G, Micheli A, Mangone L (2007). Recent cancer survival in Europe: a 2000–02 period analysis of EUROCARE-4 data. Lancet Oncol.

[4] Darmon M, Bourmaud A, Georges Q, Soares M, Jeon K, Oeyen S (2019). Changes in critically ill cancer patients' short-term outcome over the last decades: results of systematic review with meta-analysis on individual data. Intensive Care Med.

[5] Darmon M, Azoulay E, Alberti C, Fieux F, Moreau D, Le Gall JR (2002). Impact of neutropenia duration on short-term mortality in neutropenic critically ill cancer patients. Intensive Care Med.

[6] Mokart D, Textoris J, Ettori F, Chetaille B, Blache JL, Azoulay E (2011). Acute respiratory distress syndrome (ARDS) in neutropenic patients. Pulmonary involvement in patients with hematological malignancies.

[7] Azoulay E, Lemiale V, Mokart D, Pene F, Kouatchet A, Perez P (2014). Acute respiratory distress syndrome in patients with malignancies. Intensive Care Med.

[8] Azoulay E, Pickkers P, Soares M, Perner A, Rello J, Bauer PR (2017). Acute hypoxemic respiratory failure in immunocompromised patients: the Efraim multinational prospective cohort study. Intensive Care Med.

[9] Lagier D, Platon L, Chow-Chine L, Sannini A, Bisbal M, Brun JP (2016). Severity of Acute Respiratory Distress Syndrome in haematology patients: long-term impact and early predictive factors. Anaesthesia.

[10] Mokart D, Darmon M, Resche-Rigon M, Lemiale V, Pene F, Mayaux J (2015). Prognosis of neutropenic patients admitted to the intensive care unit. Intensive Care Med.

[11] van Vilet M, Verburg IW, van den Boogaard M, de Keizer NF, Peek N, Blijlevens NM (2014). Trends in admission prevalence, illness severity and survival of haematological patients treated in Dutch intensive care units. Intensive Care Med.

[12] Contejean A, Lemiale V, Resche-Rigon M, Mokart D, Pene F, Kouatchet A (2016). Increased mortality in hematological malignancy patients with acute respiratory failure from undetermined etiology: a Groupe de Recherche en Reanimation Respiratoire en Onco-Hematologique (Grrr-OH) study. Ann Intensive Care.

[13] Azoulay E, Schlemmer B (2006). Diagnostic strategy in cancer patients with acute respiratory failure. Intensive Care Med.

[14] Azoulay E, Mokart D, Kouatchet A, Demoule A, Lemiale V (2019). Acute respiratory failure in immunocompromised adults. Lancet Respir Med.

[15] Schnell D, Mayaux J, Lambert J, Roux A, Moreau AS, Zafrani L (2013). Clinical assessment for identifying causes of acute respiratory failure in cancer patients. Eur Respir J.

[16] Tolsma V, Schwebel C, Azoulay E, Darmon M, Souweine B, Vesin A (2014). Sepsis severe or septic shock: outcome according to immune status and immunodeficiency profile. Chest.

[17] Azoulay E, Mokart D, Pene F, Lambert J, Kouatchet A, Mayaux J (2013). Outcomes of critically ill patients with hematologic malignancies: prospective multicenter data from France and Belgium–a groupe de recherche respiratoire en reanimation onco-hematologique study. J Clin Oncol.

[18] Maschmeyer G, Beinert T, Buchheidt D, Cornely OA, Einsele H, Heinz W (2009). Diagnosis and antimicrobial therapy of lung infiltrates in febrile neutropenic patients: Guidelines of the infectious diseases working party of the German Society of Haematology and Oncology. Eur J Cancer.

[19] Legrand M, Max A, Peigne V, Mariotte E, Canet E, Debrumetz A (2012). Survival in neutropenic patients with severe sepsis or septic shock. Crit Care Med.

[20] Mokart D, Saillard C, Sannini A, Chow-Chine L, Brun JP, Faucher M (2014). Neutropenic cancer patients with severe sepsis: need for antibiotics in the first hour. Intensive Care Med.

[21] Mokart D, van Craenenbroeck T, Lambert J, Textoris J, Brun JP, Sannini A (2012). Prognosis of acute respiratory distress syndrome in neutropenic cancer patients. Eur Respir J.

[22] Duceau B, Picard M, Pirracchio R, Wanquet A, Pene F, Merceron S (2019). Neutropenic enterocolitis in critically ill patients: spectrum of the disease and risk of invasive fungal disease. Crit Care Med.

[23] Mokart D, Lambert J, Schnell D, Fouche L, Rabbat A, Kouatchet A (2013). Delayed intensive care unit admission is associated with increased mortality in patients with cancer with acute respiratory failure. Leuk Lymphoma.

[24] Rello J, Sarda C, Mokart D, Arvaniti K, Akova M, Tabah A (2020). Antimicrobial Stewardship in Hematological Patients at the intensive care unit: a global cross-sectional survey from the Nine-I Investigators Network. Eur J Clin Microbiol Infect Dis.

[25] Bauer PR, Chevret S, Yadav H, Mehta S, Pickkers P, Bukan RB (2019). Diagnosis and outcome of acute respiratory failure in immunocompromised patients after bronchoscopy. Eur Respir J.

[26] Pardo E, Lemiale V, Mokart D, Stoclin A, Moreau AS, Kerhuel L (2019). Invasive pulmonary aspergillosis in critically ill patients with hematological malignancies. Intensive Care Med.

[27] Legoff J, Zucman N, Lemiale V, Mokart D, Pene F, Lambert J (2019). Clinical significance of upper airway virus detection in critically ill hematology patients. Am J Respir Crit Care Med.

[28] Martin-Loeches I, Lemiale V, Geoghegan P, McMahon MA, Pickkers P, Soares M (2019). Influenza and associated co-infections in critically ill immunosuppressed patients. Crit Care.

[29] Azoulay E, Mokart D, Lambert J, Lemiale V, Rabbat A, Kouatchet A (2010). Diagnostic strategy for hematology and oncology patients with acute respiratory failure: randomized controlled trial. Am J Respir Crit Care Med.

[30] Bouteloup M, Perinel S, Bourmaud A, Azoulay E, Mokart D, Darmon M (2017). Outcomes in adult critically ill cancer patients with and without neutropenia: a systematic review and meta-analysis of the Groupe de Recherche en Reanimation Respiratoire du patient d'Onco-Hematologie (GRRR-OH). Oncotarget.

[31] Georges Q, Azoulay E, Mokart D, Soares M, Jeon K, Oeyen S (2018). Influence of neutropenia on mortality of critically ill cancer patients: results of a meta-analysis on individual data. Crit Care.

