# Supplementary material for: Acute respiratory failure in immunocompromised patients: outcome and clinical features according to neutropenia status
Source: Ann Intensive Care. 2020 Oct 22;10:146. doi: 10.1186/s13613-020-00764-7 (PMC7581668; doi:10.1186/s13613-020-00764-7)

**Additional Figure S1: Cumulative incidence of invasive mechanical ventilation during ICU stay according to neutropenia status while taking into account competing risk of mortality and discharge from ICU (Gray test: P=0.28)**


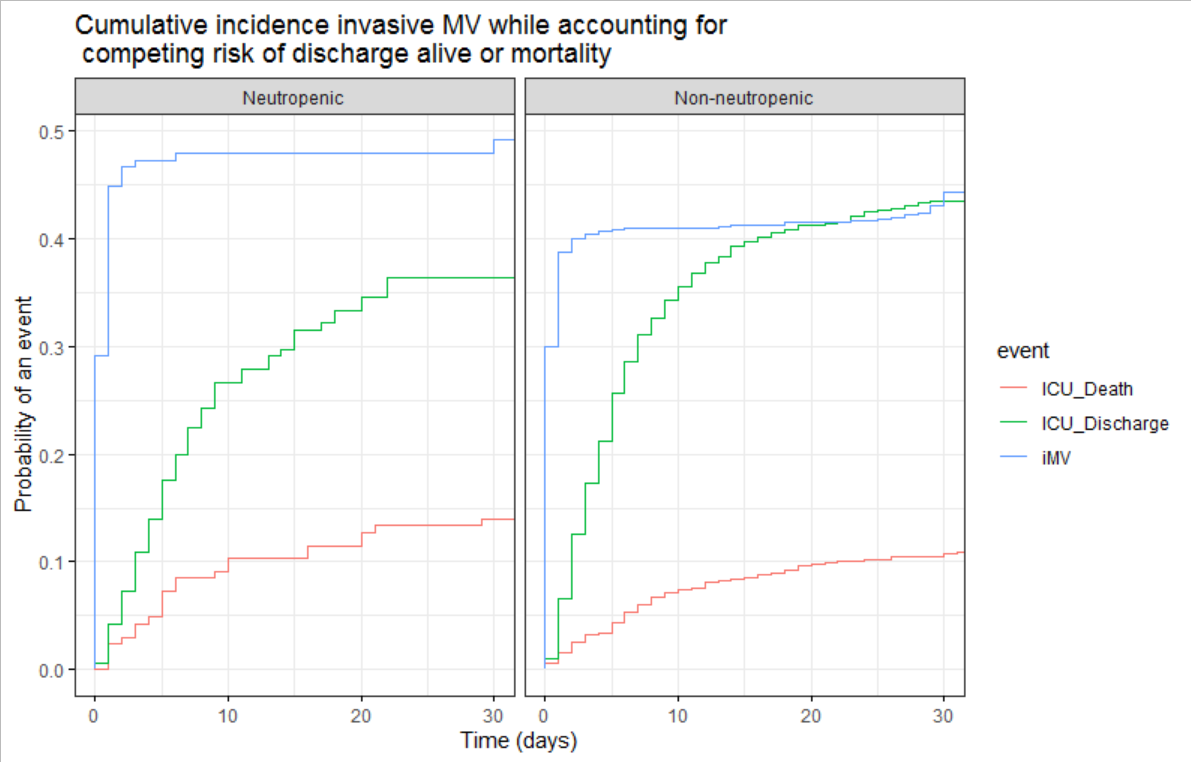

Supplement: Supplementary file 1 — Additional file 1: Fig. S1. Cumulative incidence of invasive mechanical ventilation during ICU stay according to neutropenia status while taking into account competing risk of mortality and discharge from ICU (Gray test: p = 0.28). [file 13613_2020_764_MOESM1_ESM.docx]
