# Supplementary material for: Acute respiratory failure in immunocompromised patients: outcome and clinical features according to neutropenia status
Source: Ann Intensive Care. 2020 Oct 22;10:146. doi: 10.1186/s13613-020-00764-7 (PMC7581668; doi:10.1186/s13613-020-00764-7)
Supplement: Supplementary file 2 — Additional file 2: Table S1. Final mixed selected model with neutropenia and centre effect. [file 13613_2020_764_MOESM2_ESM.docx]

**Additional Table S1: Final mixed selected model with neutropenia and centre effect**

|  | Odds Ratio (95% CI) | P value |
| --- | --- | --- |
| Age | 1.02 (1.01-1.03) | <0.001 |
| **Immunosuppression** |  |  |
| Chronic leukaemia | 0.64 (0.34-1.21) | 0.17 |
| Hodgkin disease | 1.31 (0.55-3.10) | 0.54 |
| Immunosuppressive drugs | 0.61 (0.34-1.09) | 0.09 |
| Myeloma | 0.70 (0.42-1.16) | 0.17 |
| Non Hodgkin disease | 1.39 (0.87-2.20) | 0.17 |
| Other immunosuppression | 1.01 (0.61-1.70) | 0.96 |
| Solid tumor | 1.22 (0.82-1.80) | 0.32 |
| Systemic disease | 1.02 (0.62-1.69) | 0.93 |
| **Diagnosis of acute respiratory failure** |  |  |
| Invasive fungal infection | 1.35 (0.80-2.29) | 0.27 |
| Other diagnosis | 0.87 (0.65-1.16) | 0.33 |
| Pneumocystis | 1.32 (0.68-2.58) | 0.42 |
| Unknown diagnosis | 1.50 (0.96-2.32) | 0.07 |
| Vasopressors | 2.61 (2.01-2.39) | <0.001 |
| Renal replacement therapy | 2.20 (1.57-3.07) | <0.001 |
| Neutropenia | 1.40 (0.93-2.11) | 0.11 |
