# Supplementary figures and images for: Acute respiratory failure in immunocompromised patients: outcome and clinical features according to neutropenia status
Source: Ann Intensive Care. 2020 Oct 22;10:146. doi: 10.1186/s13613-020-00764-7 (PMC7581668; doi:10.1186/s13613-020-00764-7)

**Additional Figure S2: Change in standardized mean difference after matching**


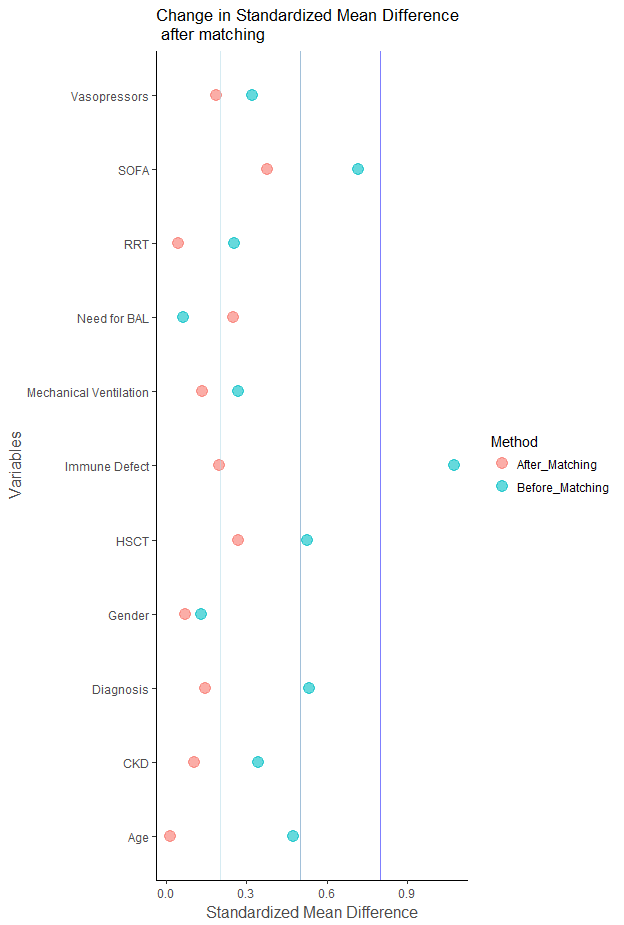

Supplement: Supplementary file 3 — Additional file 3: Fig. S2. Change in standardized mean difference after matching. [file 13613_2020_764_MOESM3_ESM.docx]
