# Supplementary material for: Acute respiratory failure in immunocompromised patients: outcome and clinical features according to neutropenia status
Source: Ann Intensive Care. 2020 Oct 22;10:146. doi: 10.1186/s13613-020-00764-7 (PMC7581668; doi:10.1186/s13613-020-00764-7)
Supplement: Supplementary file 4 — Additional file 4: Table S2. Hierarchical model assessing factors associated with hospital mortality in the matched cohort. Center effect was entered as random effect on the intercept. [file 13613_2020_764_MOESM4_ESM.docx]

**Additional Table S2: Hierarchical model assessing factors associated with hospital mortality in the matched cohort. Center effect was entered as random effect on the intercept.**

|  | Odds Ratio | 95%CI | P value |
| --- | --- | --- | --- |
| Neutropenia | 1.04 | 0.63-1.72 | 0.86 |
| SOFA | 1.21 | 1.13-1.29 | <0.001 |
| BAL not performed | 0.59 | 0.36-.97 | .038 |
| ICC Center | 0.000 |  |  |

BAL= bronchoalveolar lavage; SOFA= Sequential Organ Failure Assessment score.
