# Supplementary material for: Acute respiratory failure in immunocompromised patients: outcome and clinical features according to neutropenia status
Source: Ann Intensive Care. 2020 Oct 22;10:146. doi: 10.1186/s13613-020-00764-7 (PMC7581668; doi:10.1186/s13613-020-00764-7)

**Additional Figure S3: Main etiologies of ARF in the overall population, 1316 non-neutropenic patients vs 165 neutropenic patients**


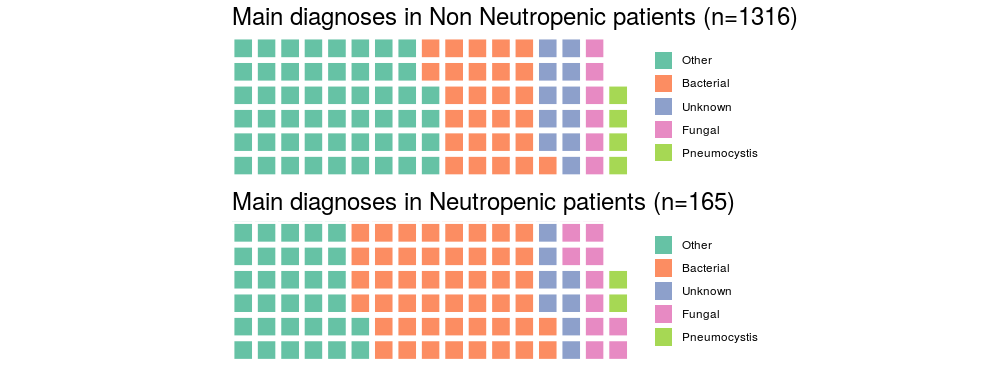

Supplement: Supplementary file 5 — Additional file 5: Fig. S3. Main etiologies of ARF in the overall population, 1316 non-neutropenic patients vs 165 neutropenic patients. [file 13613_2020_764_MOESM5_ESM.docx]
