# Supplementary material for: Acute respiratory failure in immunocompromised patients: outcome and clinical features according to neutropenia status
Source: Ann Intensive Care. 2020 Oct 22;10:146. doi: 10.1186/s13613-020-00764-7 (PMC7581668; doi:10.1186/s13613-020-00764-7)
Supplement: Supplementary file 6 — Additional file 6: Table S3. Other diagnoses (main etiologies). [file 13613_2020_764_MOESM6_ESM.docx]

**Additional Table S3: Other diagnoses (main etiologies)**

|  | **Non-neutropenic (n= 691)** | **Neutropenic (n= 53)** | **p-value** |
| --- | --- | --- | --- |
| Drug toxicity | 34 (4.9%) | 5(9.4%) | 0.27 |
| Cardiogenic pulmonary oedema | 75 (10.9%) | 13*(*24.5%) | **0.006** |
| Tumor infiltration | 114 (16.5%) | 2 (3.8%) | **0.02** |
| Aspiration pneumonia | 41 (5.9%) | 2 (3.8%) | 0.73 |
| Viral infection - Influenzae | 88 (12.7%) | 8 (15.1%) | 0.78 |
| Viral infection - Other virus | 134 (19.4%) | 15 (28.3%) | 0.17 |
| Airway obstruction | 39 (5.6%) | 1 (1.9%) | 0.39 |
